# Fatal long-term complications due to bendamustine for Waldenstrom's macroglobulinemia

**DOI:** 10.1016/j.clinsp.2025.100591

**Published:** 2025-02-06

**Authors:** Fulvio Alexandre Scorza, Carla Alexndra Scorza, Josef Finsterer

**Affiliations:** a Universidade Federal de São Paulo, Escola Paulista de Medicina (UNIFESP/EPM), São Paulo, SP, Brazil; b Neurology and Neurophysiology Center, Vienna, Austria

Waldenström's Macroglobulinemia (WM) is a clonal B-lymphocyte neoplasia, also known as lymphoplasmacytic lymphoma, with monoclonal IgM.^1^ WM manifests clinically as fatigue, lymphadenopathy, organomegaly, polyneuropathy, anemia, thrombocytopenia, hepatosplenomegaly, lymphadenopathy, amyloidosis, cryoglobulinemia and rarely hyperviscosity.^1^^,^^2^ Central Nervous System (CNS) involvement is a rare extramedullary manifestation of WM known as Bing-Neel Syndrome (BNS).^3^ WM is diagnosed by the presence of monoclonal IgM-protein in association with ≥ 10 % clonal lymphoplasmacytic cells in the bone marrow.^2^ The L265P mutation in MYD88 is detectable in >90 % of patients but is not required for diagnosis.^1^^,^^2^ Treatment is only indicated in patients with adenopathy or organomegaly, neuropathy, hyperviscosity, cryoglobulinemia, cold agglutinin disease, cytopenias, or amyloidosis.^4^ Treatment options include wait-and-see, Rituximab (RTX), bendamustine, or zanubrutinib.^1^^,^^2^ Refractory cases can be treated with bortezomib (Bortezomib), cyclophosphamide, fludarabine, thalidomide, everolimus, Bruton's tyrosine kinase inhibitors, carfilzomib, lenalidomide, and venetoclax.^1^^,^^2^^,^^5^ Predictors of outcome include age, hemoglobin level, platelet count, β2-microglobulin, LDH and monoclonal IgM levels.^1^

Bendamustine is generally well tolerated, but side effects or complications may occur in some cases. Common side effects include cough, anemia, bruising, bleeding, constipation, diarrhea, nausea, vomiting, loss of appetite, fever, fatigue, and headache.^6^^,^^7^ Renal failure is a rare complication of bendamustine.^6^ A patient with WM in whom bendamustine treatment was complicated by acute and chronic renal failure requiring long-term peritoneal dialysis and eventually leading to sepsis and death has not been reported.

The patient is a 66-year-old woman who was first diagnosed with WM with MYD88 WT at the age of 60 on the basis of the clinical picture (enlarged lymph nodes) and the bone marrow biopsy. She had already been diagnosed with Monoclonal Gammopathy of Unknown Significance (MGUS) at the age of 53. The bone marrow biopsy at the age of 59 revealed an increase in monoclonal plasma cells (IgM-lambda) with conspicuous infiltration of small B lymphocytoid cells, but the criteria for WM were not yet met. After a second bone marrow biopsy at the age of 62, which revealed lymphoplasmacytic cells, non-Hodgkin's lymphoma MYD88 74-ISSWM was diagnosed and bendamustine (50 mg/m^2^) was started at the age of 64, but had to be discontinued one month later due to acute renal failure and replaced by RTX. Due to the ineffectiveness of RTX, she was switched to ibrutinib. She has been receiving zanubrutinib since the age of 65.

Her medical history also included arterial hypertension, steatosis hepatis, osteoporosis, hyperlipidemia, hyperhomocysteinemia, nodular goiter with substituted hypothyroidism, tubulo-villous adenoma of the colon, reflux esophagitis, gastritis, renal cysts, sigmoid diverticulosis, varicose veins and cholecystolithiasis. From the age of 64, she had to undergo peritoneal dialysis due to chronic renal failure. From the same age, she developed symptoms of Restless Leg Syndrome (RLS).

At the age of 66, she was admitted due to diarrhea for two months, loss of appetite for three weeks, abdominal pain, nausea and vomiting as well as dizziness for three days. Peritonitis, gastroduodenitis, hepatopathy, thrush esophagitis and sepsis due to E. coli were diagnosed, zanubrutinib was discontinued and ceftazidine, vancomycin, fluconazole (Diflucan) and pantoprazole were started. Twelve days after admission, the patient suffered sudden cardiopulmonary arrest (Pulseless Electrical Activity [PEA]), but was successfully resuscitated with Recurrence of Spontaneous Circulation (ROSC) five minutes after starting the Cardiopulmonary Resuscitation (CPR). She was transferred to the ICU and peritoneal dialysis was switched to hemofiltration. The peritoneal dialysis catheter was removed. Evaluation for PEA revealed arterial hypotension, atrial fibrillation, left anterior hemiblock, impaired systolic function, pericardial effusion, and marked thickening of the left ventricular myocardium suggestive of amyloidosis or hypertrophic cardiomyopathy (Fig. 1). Meropenem and linezolid were administered. Despite these measures, leukocyte, CRP and procalcitonin levels were repeatedly elevated.

**Fig. 1:**
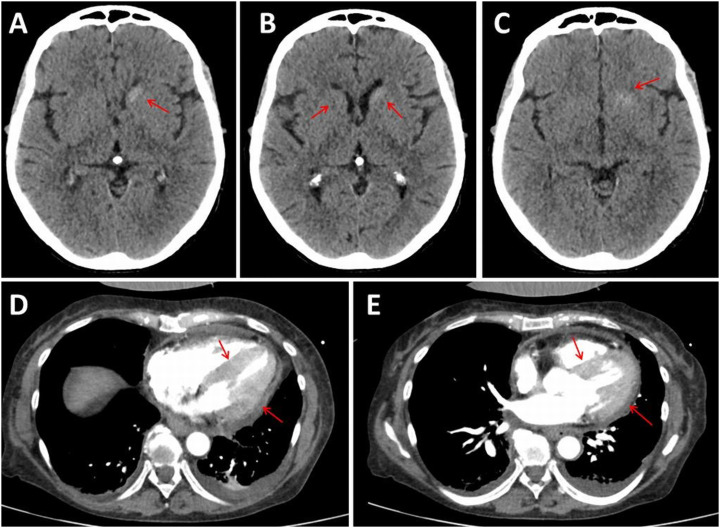
Cerebral CT (axial view) showing mild hyperdensities lateral and caudal to the left lateral ventricle/caput nuclei caudati ‒ possibly representing calcifications, hemorrhage or CNS involvement in WM (BNS), without significant change compared to a previous finding (panels A, B, C). Thoracic CT showed marked thickening of the left ventricular myocardium and pericardial effusion (panels D, E).

Clinical neurological examination for RLS revealed attention deficit, word-finding difficulties, memory impairment, anosmia, hypogeusia, anisocoria with dilated and unresponsive pupil on the left side, slight bulbar bulging on the left side, terminal ataxia, reduced (upper extremity) or absent (lower extremity) tendon reflexes, leg edema and trophic disorders. Rotigotine was prescribed and a cerebral MRI and nerve-conduction studies were ordered. A cerebral CT scan revealed bilateral caudate nucleus hyperdensities (Fig. 1). Despite maximal cardiac, renal, endocrine, immunological, anti-infectious and psychological therapy, the condition deteriorated, capillary leak syndrome persisted and heart failure required maximal adrenergic support. One month after admission, the patient received a DNR order that included discontinuation of hemofiltration and initiation of a hydromorphone perfusor. The patient died a few hours later.

The patient presented is interesting in several respects. First, administration of bendamustine resulted in acute/chronic renal failure without renal involvement in WM. Although renal involvement in WM has been repeatedly reported^8^ and although renal failure may be a rare complication of bendamustine,^9^ renal insufficiency due to bendamustine has not been reported in a WM patient. In general, renal failure occurs in approximately 1 % of patients receiving bednamustine.^9^ The mechanism of how bendamustine damages the kidney is unclear, but the damage appears to be irreversible. In the index patient, the severity of renal failure progressed, eventually leading to the need for dialysis. Second, the CCT showed a mildly hyperdense caudate nucleus, which was interpreted as calcifications, haemorrhages, or CNS involvement in WM. CNS involvement in WM has been repeatedly reported^10^ and manifests as cerebral masses. Third, WM in the index patient also manifested in the myocardium, presumably as amyloidosis, and in the peripheral nerves as neuropathy. It has been previously reported that WM can be complicated by cardiac amyloidosis and polyneuropathy. Fourth, WM was preceded by MGUS, but the patient was not tested for the MYD88 L265P variant at that time before WM was diagnosed by bone marrow biopsy.

In summary, this case demonstrates that bendamustine in WM can be complicated by progressive renal failure causing dialysis, peritonitis, and fatal sepsis due to a peritoneal dialysis catheter. Whenever possible, patients with WM requiring dialysis should undergo hemodialysis rather than peritoneal dialysis. Patients with MGUS should be screened for the pathogenic MYD88 variant in order not to miss the development of WM and appropriate treatment.

## Data access statement

All data are available from the corresponding author.

## Ethics statement

Not applicable.

## Compliance with ethics guidelines

This article is based on previously conducted studies and does not contain any new studies with human participants or animals performed by any of the authors.

## Authors’ contributions

JF: Design, data generation, literature search, discussion, first draft, critical comments, final approval. CS+FS: literature search, discussion, final approval.

## Declaration of competing interest

The author declares that the research was conducted in the absence of any commercial or financial relationships that could be construed as a potential conflict of interest.
